# Characterization and Association of Rips Repertoire to Host Range of Novel *Ralstonia solanacearum* Strains by In Silico Approaches

**DOI:** 10.3390/microorganisms11040954

**Published:** 2023-04-06

**Authors:** Juan Carlos Ariute, Andrei Giachetto Felice, Siomar Soares, Marco Aurélio Siqueira da Gama, Elineide Barbosa de Souza, Vasco Azevedo, Bertram Brenig, Flávia Aburjaile, Ana Maria Benko-Iseppon

**Affiliations:** 1 Preventive Veterinary Medicine Departament, Veterinary School, Universidade Federal de Minas Gerais, Belo Horizonte 31270-901, Minas Gerais, Brazil; 2 Genetics Department, Universidade Federal de Pernambuco, Recife 50740-600, Pernambuco, Brazil; 3 Institute of Biological and Natural Sciences, Universidade Federal do Triângulo Mineiro, Uberaba 38025-180, Minas Gerais, Brazil; 4 Department of Agronomy, Universidade Federal Rural de Pernambuco, Recife 52171-900, Pernambuco, Brazil; 5 Genetics, Ecology and Evolution Department, Universidade Federal de Minas Gerais, Belo Horizonte 31270-901, Minas Gerais, Brazil; 6 Institute of Veterinary Medicine, University Göttingen, 37077 Göttingen, Germany

**Keywords:** bioinformatics, agronomy, phythopathogens, genomic taxonomy, T3Es, host specificity

## Abstract

*Ralstonia solanacearum* species complex (RSSC) cause several phytobacteriosis in many economically important crops around the globe, especially in the tropics. In Brazil, phylotypes I and II cause bacterial wilt (BW) and are indistinguishable by classical microbiological and phytopathological methods, while Moko disease is caused only by phylotype II strains. Type III effectors of RSSC (Rips) are key molecular actors regarding pathogenesis and are associated with specificity to some hosts. In this study, we sequenced and characterized 14 newly RSSC isolates from Brazil’s Northern and Northeastern regions, including BW and Moko ecotypes. Virulence and resistance sequences were annotated, and the Rips repertoire was predicted. Confirming previous studies, RSSC pangenome is open as α≅0.77. Genomic information regarding these isolates matches those for *R. solanacearum* in NCBI. All of them fit in phylotype II with a similarity above 96%, with five isolates in phylotype IIB and nine in phylotype IIA. Almost all *R. solanacearum* genomes in NCBI are actually from other species in RSSC. Rips repertoire of Moko IIB was more homogeneous, except for isolate B4, which presented ten non-shared Rips. Rips repertoire of phylotype IIA was more diverse in both Moko and BW, with 43 common shared Rips among all 14 isolates. New BW isolates shared more Rips with Moko IIA and Moko IIB than with other public BW genome isolates from Brazil. Rips not shared with other isolates might contribute to individual virulence, but commonly shared Rips are good avirulence candidates. The high number of Rips shared by new Moko and BW isolates suggests they are actually Moko isolates infecting solanaceous hosts. Finally, infection assays and Rips expression on different hosts are needed to better elucidate the association between Rips repertoire and host specificities.

## 1. Introduction

Per year, around 20% of yield losses are due to infection by soil borne microbes [1,2]. Bacterial wilt, caused by *Ralstonia solanacearum* species complex (RSSC), is a cosmopolitan phytobacteriosis of difficult management and control in the field. It is responsible for significant yield losses in many crops in tropical regions and worldwide, affecting potato, tomato, eggplant, peppers, banana, eucalyptus, and ginger, among others [1,3,4,5].

In the state of Pernambuco, Brazil, this bacterium was detected in all mesoregions, being responsible for total loss in crops where the disease was identified [6,7]. In the last decade, many phylogenetic studies proposed the reclassification of RSSC into three distinct species according to their phylotype position and center of origins: *R. pseudosolanacearum* (phylotypes I and III, from Asia and Africa), *R. solanacearum* (phylotypes IIA and IIB, from America), and *R. syzygii* (phylotype IV, from Indonesia) [8,9,10]. Due to the broad range of hosts of RSSC, they’re commonly described in ecotypes according to the infected host and disease caused. All species in RSSC cause bacterial wilt (BW). Moko disease of Musa, brown rot of potato, and non-pathogenic to banana (NPB) are caused only by *R. solanacearum*, with brown rot and NPB being positioned in IIB as recent *R. solanacearum* strains derived from Moko. In turn, Sumatra disease of clove and blood disease bacterium (BDB) are caused by *R. syzygii* [8,11]. In Brazil, no occurrences of *R. syzygii* have been reported, whereas *R. pseudosolanacearum* and *R. solanacearum* are pointed out as being responsible for all BW cases [11]. Furthermore, Moko disease is highly prevalent in Latin America, considered an A2-level quarantine disease in the Northern (Amazonas, Pará, Rondônia, and Roraima) and Northeastern (Pernambuco and Sergipe) regions [12]. However, no phenotypic characteristics or symptoms displayed by infected plants enable distinguishing RSSC in phylotypes.

A relevant molecular mechanism in RSSC related to pathogenicity and virulence is their type III effectors (T3Es), commonly referred to as *Ralstonia* injected proteins (Rips). Those proteins are essential in pathogenicity success because they interfere with plant basal immunity and act on specific targets within cascade reactions in the cell, eliciting or attenuating hypersensitive responses [13]. Effectors triggering hypersensitive responses are related to avirulence traits, while effectors eliciting immune responses are related to virulence traits. Moreover, a series of studies has been carried out to identify the role of those effectors in host infection success. For instance, the absence of RipAA and RipP1 are linked to infection success in tobacco [14], RipS1 is linked to virulence contribution in African daisy and eggplant [15,16], and RipAZ1 is linked to avirulence in black nightshade plants [17]. Only 16% of the currently known Rips subfamilies compose the core effectome of RSSC [13,18]. Therefore, Rips repertoire tends to vary significantly with isolates’ phylotype, ecotype, and local area of occurrence.

In this study, we aimed to apply *in silico* approaches to predict the pangenome and identify the exact taxonomy of RSSC isolates causing Moko disease and BW in Brazil’s Northern and Northeastern regions, as well as investigate resistance and virulence genes, predict their Rips repertoire, and compare with previously identified Rip candidates related to host specificity.

## 2. Materials and Methods

### 2.1. Genomes Database

In total, 120 complete genomes were used for this study, 118 of *R. solanacearum* and 2 genomes of *R. pseudosolanacearum*; 106 of them were retrieved from the public genome repository of the National Center for Biotechnology (NCBI) and 14 unpublished private genomes were isolated from the Northern and Northeastern regions of Brazil, from which 12 cause Moko disease and 2 cause BW in tomato. These 14 isolates were previously sequenced on the Illumina Hi-Seq 2500 platform in a paired-end library of 2 × 150 bp at the University of Göttingen (Germany).

### 2.2. Quality Control and Assembly

The sequencing quality was estimated using the FastQC (v0.11.8) metrics report [19]. Subsequently, we used SPAdes (v3.14) [20] for genome assembly with default parameters. For genome completeness verification and assembly parameters, assembled genomes underwent evaluation through BUSCO (v4.1.2) (Benchmarking Universal Single-Copy Orthologs) [21] against the bacteria_odb10 database. Assemblies with completeness below 90% were discarded. In parallel, we also used QUAST (v5.2.0) (Quality Assessment Tool for Genome Assemblies) [22] with default parameters. The information regarding the strain name, collection site, host, and disease caused for all genomes used is available in Table A1.

### 2.3. Genome Annotation and T3Es Recovery

All genomes underwent automatic annotation through Prokka (v1.13.4) [23], a specific annotation tool for prokaryotes, to identify coding sequences (CDS) and non-coding RNAs using default parameters and databases. Further, we used PanViTa (https://github.com/dlnrodrigues/panvita, accessed on 15 December 2022) [24] to predict virulence and metal resistance genes for all 120 genomes, using VFDB [25] and BacMet [26] databases. Finally, Rips were predicted for the new 14 isolates plus 2 public genomes of Brazilian *R. solanacearum* BW isolates using the RalstoT3E’s database (https://iant.toulouse.inra.fr/bacteria/annotation/site/prj/T3Ev3/, accessed on 16 February 2022) [27] with default parameters. In this step, we only considered Rips with at least one copy in one of the isolates. From the Rips repertoire prediction, we tried to find candidate Rips for host specificity in each ecotype and compared them to previously found candidates for Moko disease [28]: RipAA, RipAB, RipAC, RipAD, RipAE, RipAI, RipAN, RipAO, RipAP, RipAU, RipAY, RipB, RipC1, RipD, RipE2, RipF1, RipG2, RipG3, RipG6, RipH1, RipH2, RipP, RipV1, and RipW. For visualization and comparison, both the absence–presence heatmaps and Venn diagrams were plotted using R standard packages.

### 2.4. Prediction of the RSSC Pangenome

To identify clusters of similar genomes, the RSSC pangenome was estimated with Roary (v3.13.0) [29] with subsequent visualization of the matrix and phylogenetic tree on Phandango (https://jameshadfield.github.io/phandango/#/main, accessed on 6 June 2022) [30] and Roary’s built-in R script. A phylogenomic tree inferred on single-copy orthologs was plotted using OrthoFinder (v2.5.4) and iTOL (v6.0) [31,32]. The pangenome’s alpha value was calculated with an *in-house* script using OrthoFinder’s outputs with a formula based on the Heap’s Law model, in which α < 1 indicates an open pangenome [33,34]:

(1) $$n=k\times N_{\gamma }$$

In which: *n* = number of genes, *N* = number of genomes, and *k* and γ are constants defined to fit the specific curve. Following, γ can be calculated as:

(2) α=1−γ

By that, α < 1 indicates an open pangenome, in which the more genomes are sequenced and added to the analysis, the more genes will be discovered. On the other hand, α > 1 indicates a closed pangenome, meaning despite more genomes being added, no significant increase in new genes would be discovered. Additionally, we also used the Least Squares Fit Principle to predict the number of singletons added to each genome and a probable number of genes for core genome stabilization, following:

(3) $$n=k\times e^{\left(x-\tau \right)}+tg\theta$$

In which: *n* = number of genes, *x* = number of genomes, *e* is the Euler number, and *k*, τ and *tg*Θ are constants.

### 2.5. Whole-Genome Methods for Taxonomy Insights

Furthermore, all genomes underwent two distinct approaches for species classification: first, an Average Nucleotide Identity (ANI) analysis was conducted through the MUMmer alignment method using pyANI (v3.0) [35], considering a ≥96% similarity criteria for different genomes belonging to the same species. Afterward, an in silico DDH (DNA–DNA hybridization) analysis was performed in the GGDC web server (v3.0) [36,37] (https://ggdc.dsmz.de/ggdc.php#, accessed on 30 June 2022), with subsequent visualization in Morpheus (https://software.broadinstitute.org/morpheus/, accessed on 8 January 2022). Due to the limitation in the number of genomes allowed in GGDC web server, we only used 77 of 120 genomes from the database, including the 14 newly sequenced ones. A ≥70% similarity criterion was considered for different genomes belonging to the same species, and ≥79–80% similarity criterion for subspecies classification [38].

## 3. Results

### 3.1. Genome Sequencing and Characterization of New Brazilian RSSC Isolates

**Figure 1:**
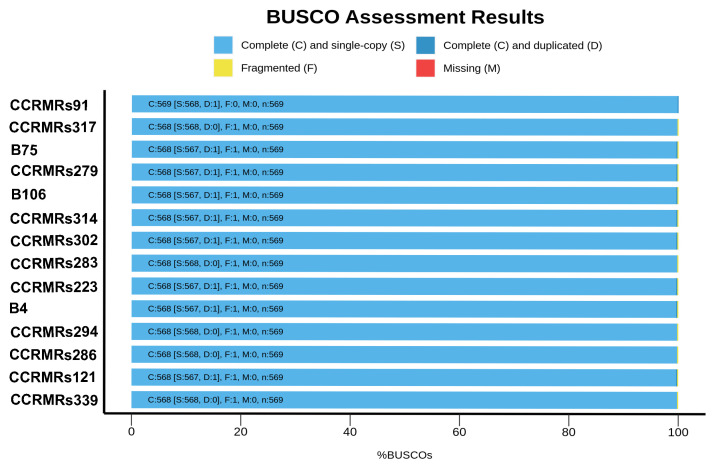
Genome completeness for Northern and Northeastern sequenced RSSC isolates.

The QUAST report revealed that the isolates B4 and CCRMRs121 had the largest and smallest genomes, with 5,858,492 and 5,364,378 base pairs, respectively. All genome sizes were similar to the average genome size for *R. solanacearum* at NCBI (5,059,182 bp). Other quality metrics for all isolates are available in Table 1. Furthermore, the BUSCO report revealed all genomes were complete considering single-copy orthologous genes. None of the assembled genomes had missing genes from the database. However, most had at least one fragmented or duplicated gene, which did not interfere with further analyses (Figure 1).

**Figure 2:**
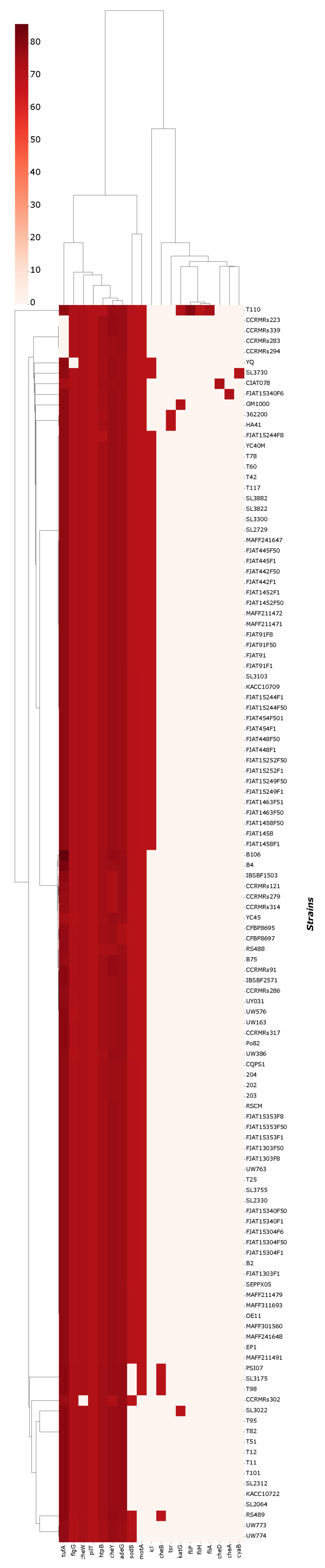
Clustermap for virulence genes presence and similarity against VFDB.

**Figure 3:**
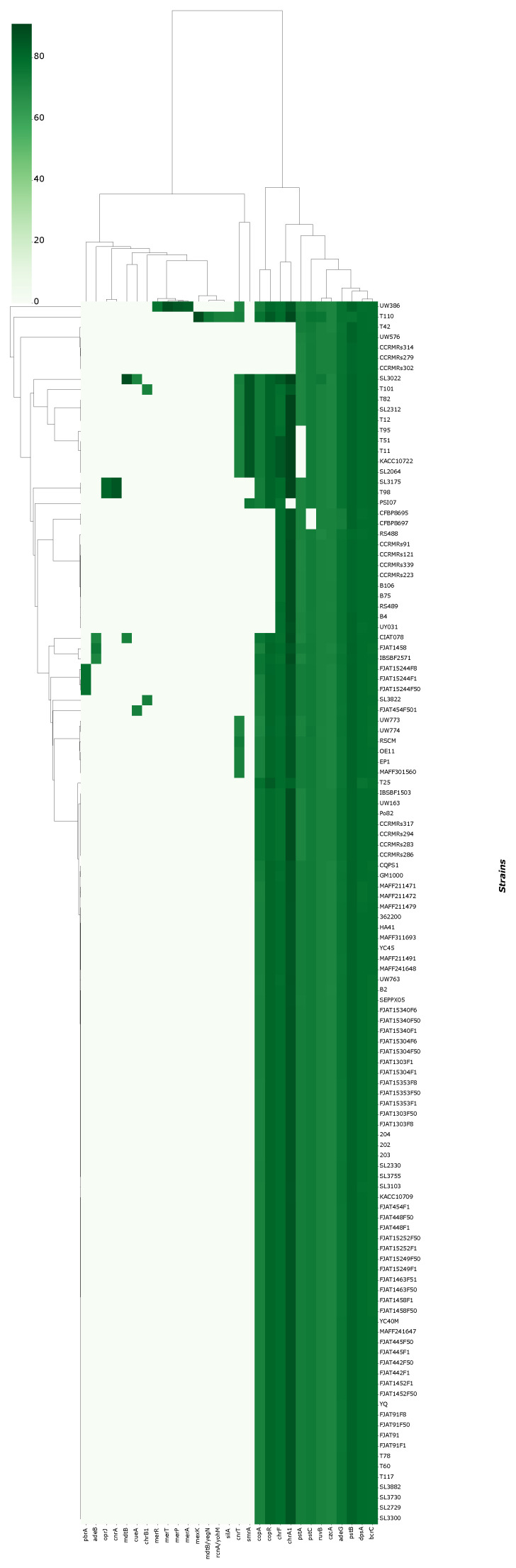
Clustermap for metal resistance genes presence and similarity against BacMet database.

Genomes presented between 5034 and 4592 coding sequences and 61 to 66 non-coding RNAs (including tRNAs, rRNAs, tmRNAs, and others). According to NCBI, the average count of coding sequences in *R. solanacearum* is 4774. No direct correlation was found between CDS-ncRNA amounts and the type of disease caused by each isolate. Overall, 19 virulence genes (*ade*G, *che*A, *che*B, *che*D, *che*W, *che*Y, *cya*B, *flg*G, *fli*A, *fli*M, *fli*P, *htp*B, *icl*, *kat*G, *mot*A, *pil*T, *sod*B, *tsr*, *tuf*A, and *mot*A) and 29 metal resistance genes (*ade*B, *ade*G, *bcr*C, *chr*A1, *chr*B1, *chr*F, *cnr*A, *cnr*T, *cop*A, *cop*R, *cue*A, *czc*A, *dps*A, *mdt*B, *mdt*B/*yeg*N, *mer*A, *mer*P, *mer*R, *mer*T, *mex*K, *opr*J, *pbr*A, *pst*A, *pst*B, *pst*C, *rcn*A/*yoh*M, *ruv*B, *sil*A, and *smr*A) were predicted for the isolates. All predicted genes had a similarity of ≥60%. The most abundant genes were *ade*G, *che*Y, *htp*B, and *pil*T, present in 118 of 120 genomes. Similarly, for metal resistance genes, the most abundant were *ade*G, *bcr*C, *czc*A, *dps*A, *pst*B, and *ruv*B. Strains UW386, T110, and SL3022, isolated from soil and infected potato, exhibited the most quantity of metal resistance genes, with 17 and 16, respectively. In parallel, strains T110 and SL3730, exhibited the highest amounts of virulence genes, although all genomes presented 10 to 7 virulence genes. The newly sequenced genomes presented 9 to 7 virulence genes and 10 to 8 metal resistance genes. Neither host/source nor isolate origins seemed to have a correlation to genetic virulence of resistance. The clustermaps for VFDB and BacMet are available in Figure 2 and Figure 3.

### 3.2. RSSC Pangenome and Genomic Taxonomy of Newly Sequenced *R. solanacearum* Isolates

**Figure 4:**
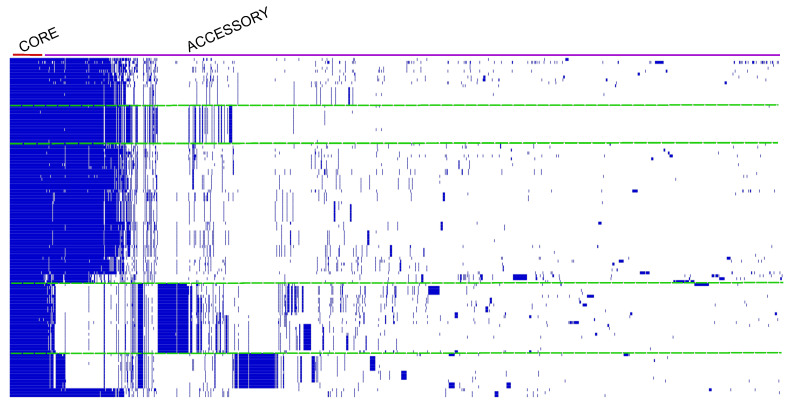
*R. solanacearum* species complex (RSSC) pangenome profile. From left to right, blue regions homogeneously distributed represent the core genome, while blue spots represent the unique genome. Blocks underneath red line make up the core genome, whilst blocks underneath purple line make up the accessory genome. From the presence–absence profile, it is possible to identify 4 major patterns in the pangenome profile, delimited by the green dotted lines.

**Figure 5:**
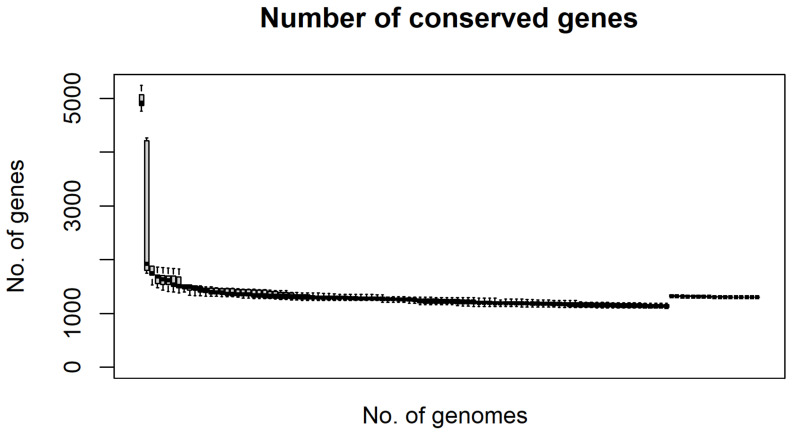
Pangenome development considering conserved genes throughout the 120 genomes.

A total of 29,507 genes were predicted for the RSSC pangenome, of which 22,002 are unique genes, 6040 are in the accessory genome, and 1465 are in the core pangenome. The predicted pangenome clusterized the 120 genomes in at least four different groups, which match the phylotype classification of RSSC based on their centers of origin and phylotypes (Figure 4). Applying the OrthoFinder results to Heap’s Law, $\;n≅4517.340\times n^{\left(0.223\right)}$ resulted in 12,094 ortholog families in the pangenome, with α≅0.77 indicating an open pangenome. Moreover, the value of tgΘ the Least Squares Fit Principle revealed that 2883 ortholog families compose the RSSC core genome (as n=1663.212×exp[−x/444.977]+1561.917), and 28 ortholog families are strain-specific (as n=90.024×exp[−x/101.874]+0.1905). By that, we predict that at each new genome added to the RSSC pangenome, ≅0.1905 new ortholog genes would be discovered, and the core genome would stabilize in around 1562 genes. Overall, the RSSC pangenome tends to stabilize once a steady low number of conserved genes was maintained through all genomes (Figure 5).

**Figure 6:**
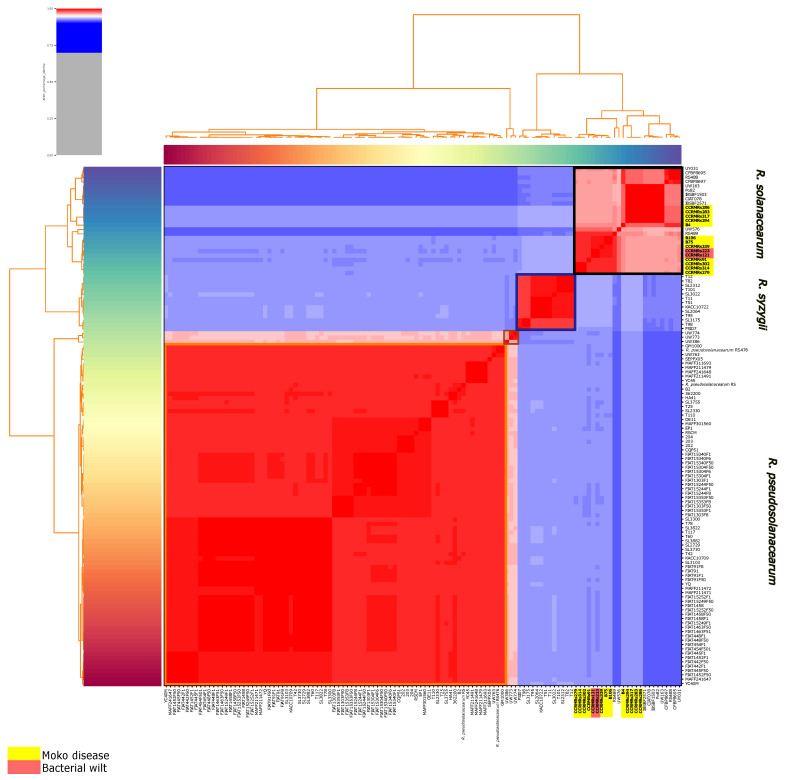
ANI analysis clustermap of public *R. solanacearum* genomes on NCBI and 14 new Brazilian *R. solanacearum* genomes. Upwards, there are phylotypes I (orange box), III (brown box), IV (blue box), IIA, and IIB clusters (black box), composing *R. pseudosolanacearum*, *R. syzygii*, and *R. solanacearum*, respectively. Newly sequenced genomes are in bold and highlighted in red and yellow according to their respective ecotypes.

**Figure 7:**
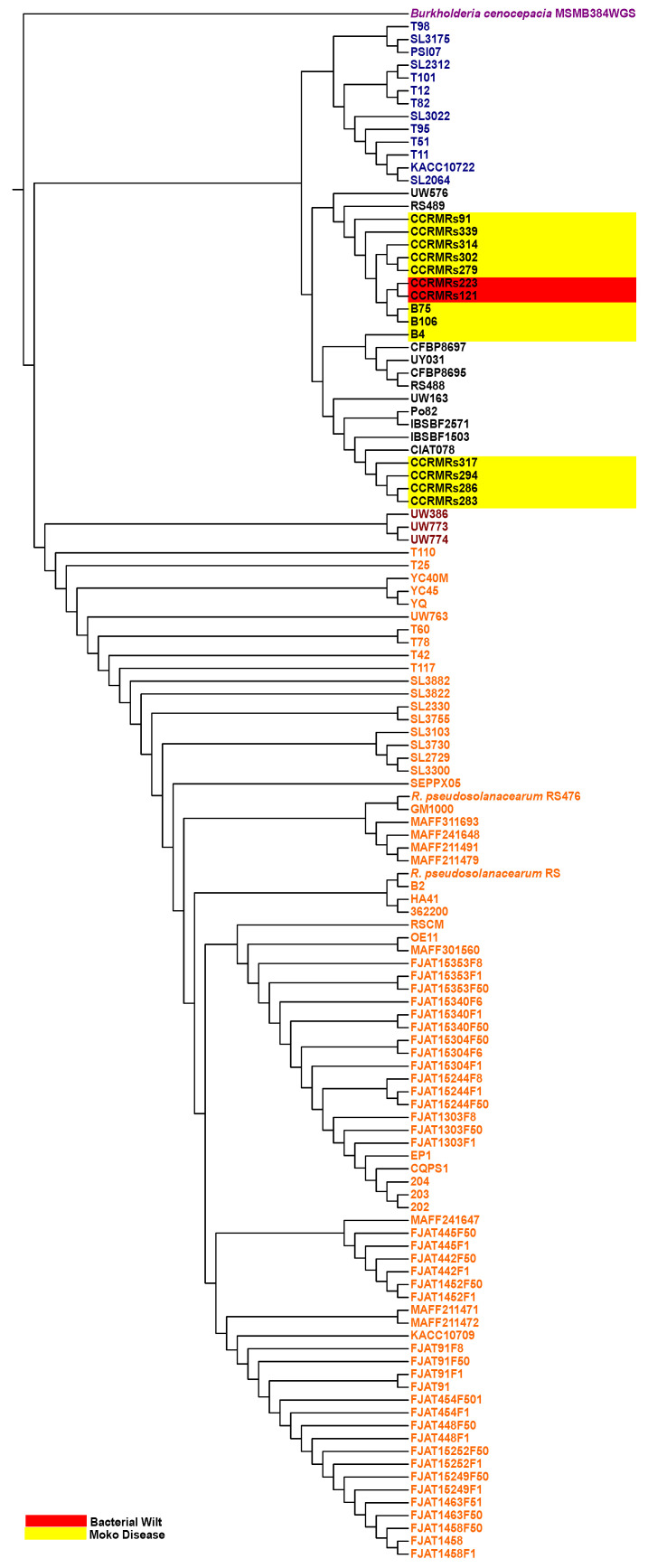
Phylogenomic tree of *R. solanacearum* species complex (RSSC) strains used in this work. Strains’ names are colored according to Figure 6 pattern: phylotypes I in orange, phylotypes III in brown, phylotypes IV in blue, and phylotypes II in black. Newly sequenced genomes are also highlighted according to their respective ecotypes.

In ANI analysis, three groups of genomes were formed, separated by similarity below 90% (Figure 6). First, *R. solanacearum* genomes from public databases isolated in China and Japan clustered with *R. pseudosolanacearum* genomes isolated in Brazil, indicating they all belong to phylotype I. Next, in phylotype III, three *R. solanacearum* genomes isolated in Africa composed a much smaller cluster relatively similar to the previous one, indicating they’re also *R. pseudosolanacearum* genomes. The following cluster was composed of *R. solanacearum* genomes isolated in South Korea, but since they were not similar to the previous or next cluster, they make up phylotype IV or *R. syzygii*. None of the new isolates clustered within the clusters mentioned up to now. The reminiscent cluster was composed of American isolates, except for CFBP strains from Iran. Among this last cluster, there was a clear division of more similar genomes: isolates CCRMRs283, CCRMRs286, CCRMRs294, and CCRMRs317 formed a smaller cluster (Cluster 1), and B75, B106, CCRMRs91, CCRMRs121, CCRMRs223, CCRMRs279, CCRMRs302, CCRMRs314, and CCRMRs339 formed a more significant cluster (Cluster 2). The genome from isolate B4 was the most distinct of all 14 but still more similar to Cluster 1 than Cluster 2 genomes. The new isolates present in Cluster 1 caused only Moko disease, while public genomes in it were obtained from isolates of other ecotypes, including NPB and brown rot. New isolates present in Cluster 2 caused Moko disease or BW on tomato only, indicating they are, respectively, phylotypes IIB and IIA, and part of the actual *R. solanacearum* species. This distribution of strains was also true for the phylogenomic tree based on gene family conservation found on OrthoFinder (Figure 7); however, it is possible to notice that isolate B4 was more distant from other IIB isolates than in ANI profile, clustering a clade with CFBP8695, CFBP8697, RS488, UY031, and UW163. That being the case, all sequenced isolates causing BW in this study are phylotype II strains.

**Figure 8:**
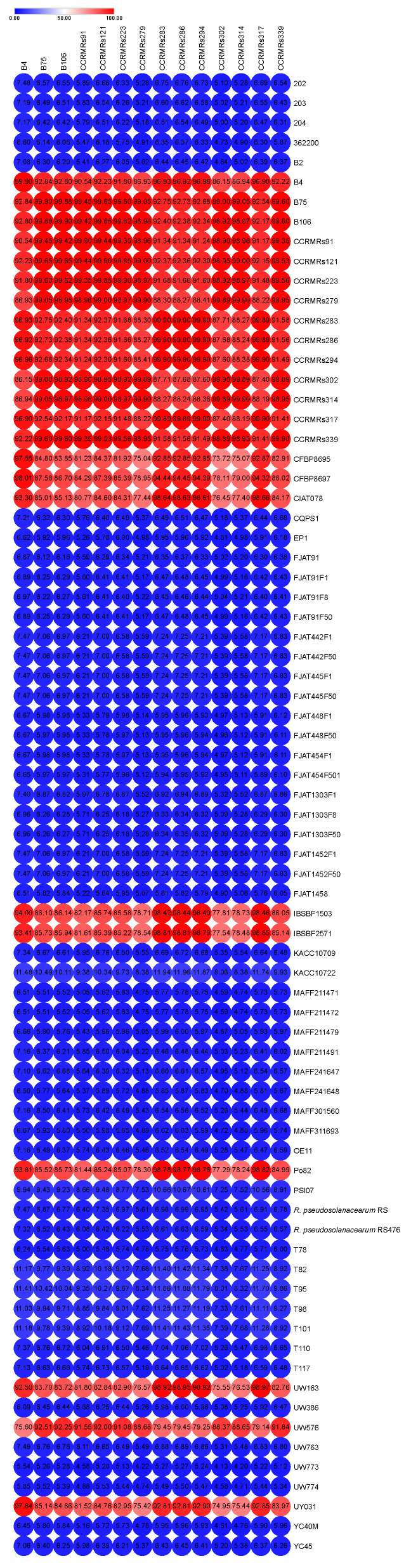
Heatmap representing the in silico DNA–DNA hybridization (*is*DDH) of genomes from *R. solanacearum* species complex (RSSC). The value for each DDH is available inside each dot.

Even though the ANI analysis and phylogenomic tree evidence two distinct clades within the newly sequenced isolates, the in silico DDH values varied significantly from pairwise comparison, and it was not possible to find a consensus that separated IIA and IIB isolates in subspecies (Figure 8).

### 3.3. Prediction of Rips Repertoire of *R. solanacearum* Strains and Ecotype Correlation

**Figure 9:**
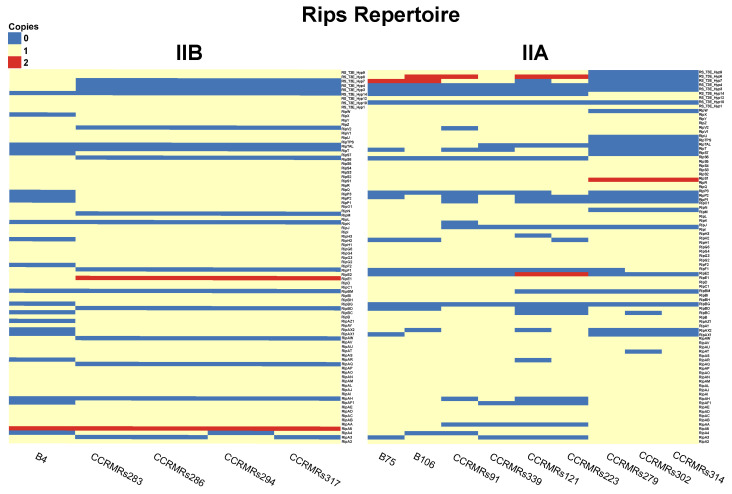
Heatmap of Rips repertoire of each new Brazilian *R. solanacearum* isolates IIB (**left**) and IIA (**right**).

In total, 88 subfamilies of Rips were present in the new 14 isolates. Overall, B4 and B76 had the lowest and the highest number of predicted Rips, with 67 and 76 out of 88, respectively. The Rips repertoire of each isolate is available in Figure 9.

**Figure 10:**
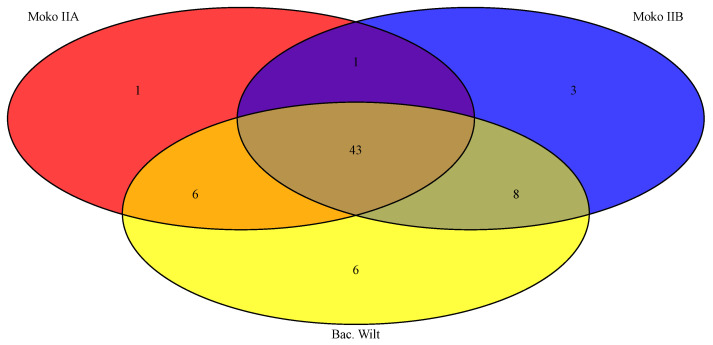
Rips shared among the sequenced isolates used in this study only.

**Figure 11:**
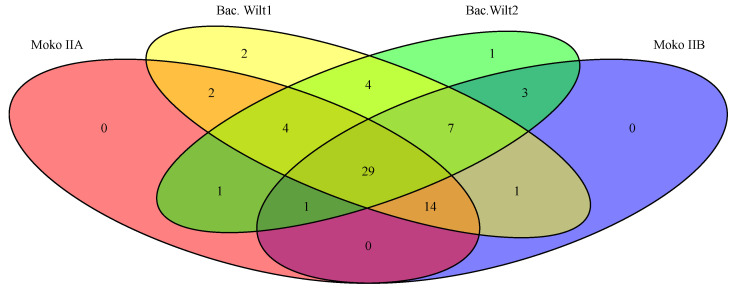
Rips shared among newly sequenced isolates (Moko IIA, Moko IIB, and BW1) plus public Brazilian BW isolates, RS488 and RS489 (BW2).

**Figure A1:**
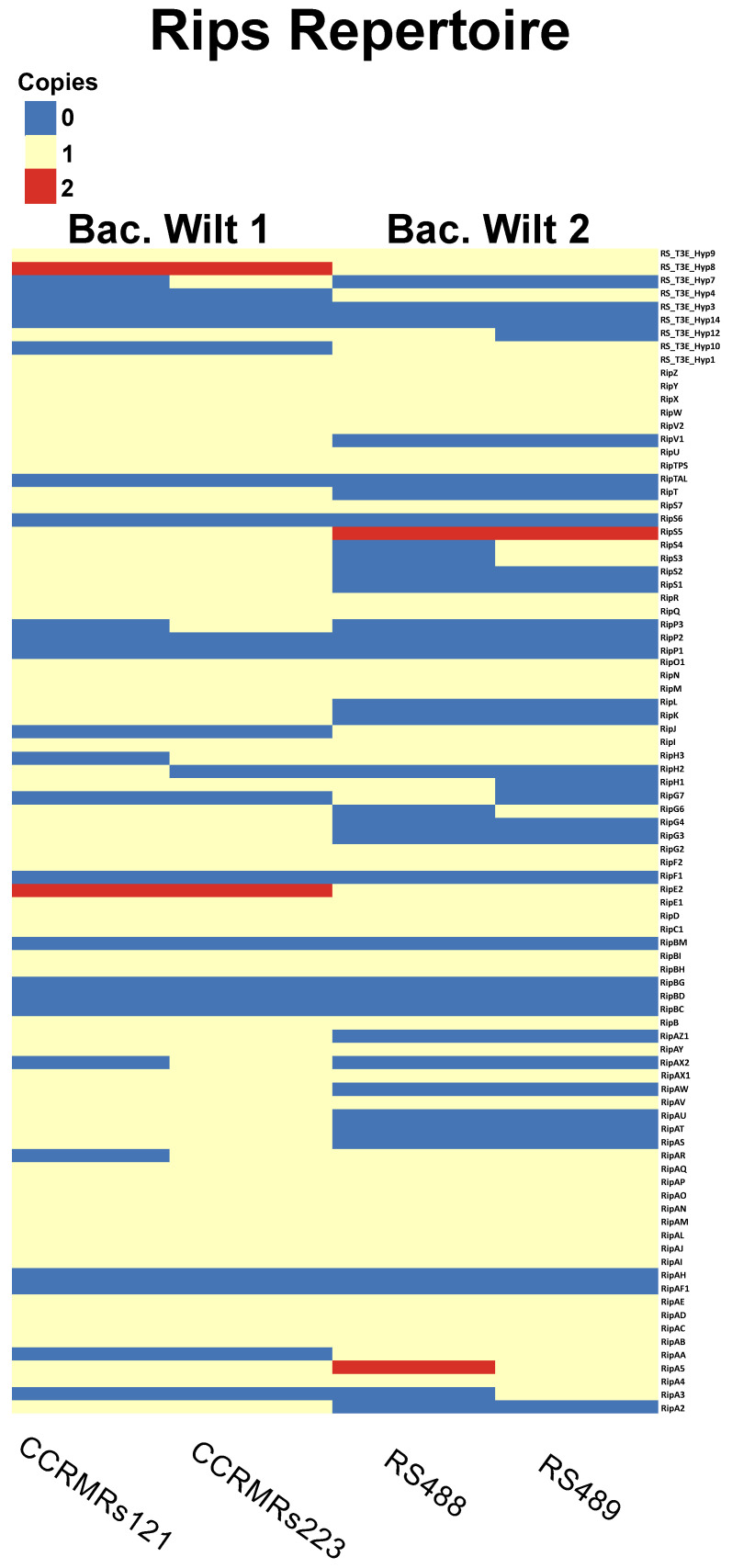
Heatmap of Rips repertoire of newly sequenced (Bac. Wilt 1) and publicly available (Bac. Wilt 2) genomes from isolates causing bacterial wilt in Brazil. The genomes on the right have notably fewer Rips than the newly sequenced ones, despite all causing diseases in tomato.

Cluster IIB Rips repertoire was more homogeneous than Cluster IIA’s, despite a higher number of isolates causing Moko in the latter. Considering both clusters, only six events of Rip duplication occurred: RipA5, RipEI1, RipE2, RipS1, RS_T3E_Hyp7, and RS_T3E_Hyp8. There was no duplication event in common for both Clusters, and there was no Rip absent in all 14 isolates besides the hypothetical ones. Congruently with the profile observed in ANI, B4 also exhibited the most distinct pattern of Rips presence–absence–duplication compared to the other 13 isolates. Starting with Cluster IIB, they commonly shared 55 Rips, and B4 presented ten exclusive Rips: RipAQ, RipAW, RipBD, RipF1, RipM, RipS6, RipV2, RS_T3E_Hyp3, RS_T3E_Hyp4, and RS_T3E_Hyp7. All isolates shared a duplication of RipA5; however, only B4 did not share a duplication of RipE1, and B4 and CCRMRs294 shared an absence of RipA4. In Cluster IIA, isolates causing Moko disease commonly shared 51 Rips. The isolates CCRMRs279, CCRMRs302, and CCRMRs314 had remarkably similar repertoires, except for the absence of RipAT and RipBC in CCRMRs302, and the absence of RipF1 in CCRMRs279. Finally, the new BW isolates commonly shared 63 Rips. Both isolates have almost the same repertoire, except for the absence of RipAR, RipAX2, RipH3, RipP3, and RS_T3E_Hyp7 in CCRMRs121, and the absence of RipH2 in CCRMRs223. Moko IIA, Moko IIB, and BW isolates commonly shared 43 Rips. These comparisons also revealed that very few Rips were ecotype-specific; the 12 isolates causing Moko disease only commonly shared RipH3. Moko IIA isolates exclusively shared RipAR, while Moko IIB isolates exclusively shared four Rips (RipAA, RipJ, and RS_T3E_Hyp10), and BW isolates exclusively shared six Rips (RipA4, RipAX1, RipK, RipS7, RipT, and RipV2). More Rips were exclusively shared among Moko IIB and BW isolates (RipAT, RipE2, RipN, RipTPS, RipU, RipZ, RS_T3E_Hyp8, and RS_T3E_Hyp9) than among Moko IIA and BW (RipAQ, RipAW, RipAZ1, RipF2, RipM, and RipY) (Figure 10). Of the 22 candidate Rips for Moko disease suggested by Ailloud et al. [28], only 4 were not commonly shared by the 12 Moko isolates: RipAA, RipE2, and RipF1 in Moko IIA and RipF1 and RipH2 in Moko IIB. However, it is important to highlight that only three of those Moko candidate Rips were not commonly shared by BW isolates: RipF1, RipH2, and RipAA. In contrast to public genomes of other Brazilian *R. solanacearum* infecting tomato, RS488 and RS489 (BW2) shared 44 Rips with CCRMRs121 and CCRMRs223 (BW1), with 16 Rips exclusively shared by BW1. Interestingly, isolates in BW1 shared more Rips with Moko IIA and IIB isolates than BW2, resting only 29 Rips shared by all four groups from the 43 early found (Figure 11). The Rips repertoire comparison of BW1 and BW2 is available in Figure A1.

## 4. Discussion

### 4.1. Pangenome and Nucleotide Identity Analysis Reveal Global Misclassification of RSSC Isolates in Public Databases and Genetic Diversity of New Brazilian Isolates

Our study used a large dataset of high-quality publicly available *R. solanacearum* complete genomes, elucidating their taxonomy via robust in silico whole-genome approaches confirming many previous findings [9,10,35,39,40]. The misclassification of older *R. solanacearum* genomes has recently been addressed by Sharma et al. [41], who also pointed out the discrepancy in representative genomes from African and South Asian isolates available at NCBI. As the sequevar/biovar classification has been shown to fail at represent the diversity of highly recombinogenic isolates [41], whole genome methods, such as ANI, *is*DDH and phylogenomic inferences are more prone to accurately provide the genetic diversity on RSSC and other bacterial phytopathogens with controversial taxonomy [35]. The open pangenome profile observed through our analysis corroborates up-to-date studies, with similar values found for core, accessory, and unique genomes [42] (Geng et al., 2022). As soil borne microbes, the resistance of *Ralstonia* strains to heavy metals was described a long time ago [5,43,44], but no recent analyses have included Brazilian isolates. Considering that pesticides and fertilizers commonly used in high-production crops typically comprise heavy metals in their composition [45,46], these findings raise an alert for small and big producers in countries such as Brazil that struggle with *R. solanacearum* infestation. As for the new RSSC isolates from Brazil’s Northern and Northeastern regions, they fit in *R. solanacearum*, but are from separate phylotypes. The fact that 12 isolates infecting *Musa* in close geographic spots still differ in phylotype sublevel only shows how diverse Brazilian RSSC isolates are, which corroborates with the hypothesis of the Amazon region being the diversity center of phylotypes IIA and IIB [7,47]. At first look, most of the newly sequenced isolates fitting in phylotype IIA might seem a surprise, as most of the Brazilian isolates are actually included in phylotype IIB, followed by phylotypes IIA and I. However, it has also been reported that phylotype IIA isolates have a higher proportional presence in Brazil’s North and Northeastern regions. In contrast, phylotype IIB has a higher abundance in Central, Southeastern and South regions [7,48]. Since phylotype IIA isolates have been characterized as more genetically diverse and recombinant than IIB [47], a less diverse repertoire of Rips was expected for the latter, and also because at least two different ecotypes were suspected for IIA isolates. Analyzing the Rips repertoire is important because each subfamily of Rip plays distinct roles throughout the infection process, depending on the environment, tissue, and signals recognized within the host [49]. On that thought, we suggest here that Rips that were not commonly shared by all isolates of their respective ecotype might contribute to their individual virulence when infecting the host. In this sense, a good indicator is that B4 was isolated from a banana plant with more severe symptoms on roots, while all other Moko isolates were isolated from banana plants with wilted leaves and healthy roots. The presence of more than one copy of Rips and paralog subfamilies in RSSC is largely documented. Even though it has been described as genetic redundancy, it is also seen as a general strategy for giving bacterial virulence robustness via acting on similar targets, participating in the same molecular functions and biological processes [18,50].

### 4.2. Rips Repertoire of Brazilian Isolates Are More Correlated to Genomic Similarity Rather Than Ecotype

As for the duplicated Rips, RipA5 (AWR5), and RipE act as typical avirulence factors eliciting hypersensitive responses on *Arabidopsis thaliana* and *Nicotiana benthamiana* and suppressing the expression of jasmonic-acid-dependent genes and salicylic acid synthesis [51,52]; however, RipAC and RipAY inhibit RipE1-mediated HR [18]. In contrast, RipS1 acts as a virulence factor that inhibits key targets on reactive oxygen species (ROS) pathways [51], considering that alone, CCRMRs279, CCRMRs302, and CCRMRs314 would have a higher potential for more virulent behavior.

A few Rips have been correlated with host specificity in South Asian RSSC strains infecting solanaceous hosts, with RipAS3 and RipH3 linked to pathogenicity in tomato and RipAC linked to pathogenicity in eggplants [53]. However, in RS488 no copy for RipS3 was predicted (see Figure A1), even though it caused BW in tomato. Indeed, this reinforces the hypothesis that a repertoire of Rips is keener to the success of pathogenicity in some hosts than in a few groups of Rips. We suggest that the presence of previous Moko candidates was more accurate for IIB isolates because it compared different ecotypes present only in phylotype IIB, such as NPB and brown rot, and also due to their clonal behavior. Therefore, the greater difference observed in Moko IIA isolates might indicate different selective pressure on those strains derived from the higher genetic diversity observed in this phylotype, making Rips gain or loss more probable. The broad presence of Moko candidates in the new BW isolates and their broadly shared Rips repertoire can be explained by two main arguments: the first one is that the most common recent ancestor of *Ralstonia* isolates was already capable of infecting banana and host-adapted polymorphisms (HAP) would be present in the Rips derived from it, making them functionally specialized either for solanaceous and musaceous hosts respective defense mechanisms [28].

Moreover, it has been shown that NPB and Moko disease strains have minimal genomic differences and still have high gene expression differences when infecting their respective hosts [54]. Thus, even if Moko and BW isolates have no significant differences in their Rip sequences, their gene expression would still differ when infecting different hosts. The second argument is that these BW isolates are actually from Moko ecotype infecting solanaceous hosts due to the optimal environmental conditions found in Brazil’s Northeastern region, as it has been previously reported in environments with high temperatures and humidity conditions [55,56]. This argument gains strength when considering that the pan-effectome of *R. solanacearum* is clearly diverse with a small core effectome of 16 Rips [13,18] contrasting with the 43 Rips present in all 14 isolates of two different ecotypes. Moreover, their Rips repertoire was more similar with Moko isolates than with RS488 and RS489. Hence, based on what we found and considering the second argument, only 14 Rips would be eligible candidates for Moko disease: RipA2, RipAS, RipAU, RipG3, RipG4, RipG6, RipH1, RipL, RipS1, RipS2, RipS3, RipS4, and RS_T3E_Hyp12.

## 5. Conclusions

The present study is the first to include Brazilian isolates of *Ralstonia* and use a robust effector database to characterize the effectome of Brazilian isolates. The commonly shared Rips by isolates in different ecotypes might aid in further phytopathology studies by providing target avirulence proteins in hosts when searching for breeds resistant to bacterial wilt, Moko, and so on. It is important to note that further research efforts, preferably with in vitro and in vivo data on gene expression and infection essays on different hosts are required to determine whether the Rips identified here are essential candidates for ecotype specificity. In addition, more phylotype IIA isolates causing bacterial wilt in Solanaceae to identify commonly shared Rips in this ecotype are needed. Finally, even though Rips presence/absence is an excellent indication for host specificity association, it is not the final determinant. The whole-genome approaches were essential in correctly identifying these isolates’ taxonomy, proving their potential for solving complicated bacterial species complexes, such as RSSC. Efforts to characterize hypothetical and redundant Rips are essential to elucidate missing roles on molecular pathways linked to triggered and innate immunity in plants.

## Figures and Tables

**Table 1:** Quality metrics for Northern and Northeastern sequenced RSSC isolates.

Isolate	Size (Mb)	Contigs	N50	L50
B106	5.50	46	399,454	5
B4	5.85	50	574,994	5
B75	5.42	77	333,179	6
CCRMRs121	5.36	35	504,573	4
CCRMRs223	5.57	53	296,540	5
CCRMRs279	5.70	441	46,716	34
CCRMRs283	5.46	81	204,913	8
CCRMRs286	5.46	81	185,753	9
CCRMRs294	5.47	81	204,912	8
CCRMRs302	5.64	380	37,222	42
CCRMRs314	5.69	381	37,222	42
CCRMRs317	5.50	80	205,138	7
CCRMRs339	5.50	249	238,614	8
CCRMRs91	5.46	81	105,718	18

## Data Availability

All 14 newly announced sequenced genomes from Brazilian Northern and Northeastern regions are available on GenBank/NCBI under BioProject PRJNA763940 (https://www.ncbi.nlm.nih.gov/bioproject/?term=PRJNA763940, accessed on 30 January 2022). All other publicly available genomes are listed in Table A1.

## Abbreviations

The following abbreviations are used in this manuscript:

BW	Bacterial Wilt
Rips	*Ralstonia* Injected Proteins
T3Es	Type III Effectors
MDPI	Multidisciplinary Digital Publishing Institute
DOAJ	Directory of open access journals
TLA	Three letter acronym
LD	Linear dichroism

## Appendix A

**Table A1:** General information regarding genomes used in this work.

Strains	GenBank	Bioproject	Origin	Host	Disease
*R. pseudosolanacearum* RS	NZ_CP046674	PRJNA594457	China (YN)	Tobacco	Bacterial wilt
*R. pseudosolanacearum* RS476	NZ_CP021762	PRJNA388859	Brazil (MA)	Tomato	Bacterial wilt
*R. solanacearum* B106	JAIVFC000000000	PRJNA763940	Benjamin Constant, AM, BR	Banana	Moko disease
*R. solanacearum* B4	JAIVEX000000000	PRJNA763940	Anamã, AM, BR	Banana	Moko disease
*R. solanacearum* B75	JAIVFE000000000	PRJNA763940	Tefé, AM, BR	Banana	Moko disease
*R. solanacearum* CCRMRs121	JAIVEU000000000	PRJNA763940	Belém de São Francisco, PE, BR	Tomato	Bacterial wilt
*R. solanacearum* CCRMRs223	JAIVEY000000000	PRJNA763940	Bezerros, PE, BR	Tomato	Bacterial wilt
*R. solanacearum* CCRMRs279	JAIVFD000000000	PRJNA763940	Manicoré, AM, BR	Banana	Moko disease
*R. solanacearum* CCRMRs283	JAIVEZ000000000	PRJNA763940	Benjamin Constant, AM, BR	Banana	Moko disease
*R. solanacearum* CCRMRs286	JAIVEV000000000	PRJNA763940	Benjamin Constant, AM, BR	Banana	Moko disease
*R. solanacearum* CCRMRs294	JAIVEW000000000	PRJNA763940	Benjamin Constant, AM, BR	Banana	Moko disease
*R. solanacearum* CCRMRs302	JAIVFA000000000	PRJNA763940	Fonte Boa, AM, BR	Banana	Moko disease
*R. solanacearum* CCRMRs314	JAIVFB000000000	PRJNA763940	Tefé, AM, BR	Banana	Moko disease
*R. solanacearum* CCRMRs317	JAIVFF000000000	PRJNA763940	Tefé, AM, BR	Banana	Moko disease
*R. solanacearum* CCRMRs339	JAIVET000000000	PRJNA763940	Coari, AM, BR	Banana	Moko disease
*R. solanacearum* CCRMRs91	JAIVFG000000000	PRJNA763940	Igreja Nova, AL, BR	Banana	Moko disease
*R. solanacearum* 202	NZ_CP049789	PRJNA609910	China (GD)	Tobacco	Bacterial wilt
*R. solanacearum* 203	NZ_CP049791	PRJNA609906	China (GD)	Tobacco	Bacterial wilt
*R. solanacearum* 204	NZ_CP049793	PRJNA609905	China (GD)	Tobacco	Bacterial wilt
*R. solanacearum* 362200	NZ_CP065531	PRJNA668065	China (FJ)	Peanut	Bacterial wilt
*R. solanacearum* B2	NZ_CP049787	PRJNA609907	China (GD)	Tobacco	Bacterial wilt
*R. solanacearum* CFBP8695	CP047138	PRJNA596809	Iran	Potato	Bacterial wilt
*R. solanacearum* CFBP8697	CP047136	PRJNA596668	Iran	Potato	Bacterial wilt
*R. solanacearum* CIAT078	NZ_CP051295	PRJNA608676	Colombia	Plaintain	Moko disease
*R. solanacearum* CQPS1	NZ_CP016914	PRJNA331070	China (SD)	Tobacco	Bacterial wilt
*R. solanacearum* EP1	NZ_CP015115	PRJNA288736	China (GD)	Eggplant	Bacterial wilt
*R. solanacearum* FJAT1303F1	NZ_CP052128	PRJNA622642	China (FJ)	Tomato	Bacterial wilt
*R. solanacearum* FJAT1303F50	NZ_CP052126	PRJNA622642	China (FJ)	Tomato	Bacterial wilt
*R. solanacearum* FJAT1303F8	NZ_CP052130	PRJNA622642	China (FJ)	Tomato	Bacterial wilt
*R. solanacearum* FJAT1452F1	NZ_CP052124	PRJNA622642	China (FJ)	Tomato	Bacterial wilt
*R. solanacearum* FJAT1452F50	NZ_CP052122	PRJNA622642	China (FJ)	Tomato	Bacterial wilt
*R. solanacearum* FJAT1458	NZ_CP016554	PRJNA329182	China (FJ)	Tomato	Bacterial wilt
*R. solanacearum* FJAT1458F1	NZ_CP052120	PRJNA622642	China (FJ)	Tomato	Bacterial wilt
*R. solanacearum* FJAT1458F50	NZ_CP052118	PRJNA622642	China (FJ)	Tomato	Bacterial wilt
*R. solanacearum* FJAT1463F50	NZ_CP052114	PRJNA622642	China (FJ)	Tomato	Bacterial wilt
*R. solanacearum* FJAT1463F1	NZ_CP052116	PRJNA622642	China (FJ)	Tomato	Bacterial wilt
*R. solanacearum* FJAT15244F1	NZ_CP052112	PRJNA622642	China (FJ)	Tomato	Bacterial wilt
*R. solanacearum* FJAT15244F50	NZ_CP052110	PRJNA622642	China (FJ)	Tomato	Bacterial wilt
*R. solanacearum* FJAT15244F8	NZ_CP059376	PRJNA647244	China (FJ)	-	-
*R. solanacearum* FJAT15249F1	NZ_CP052108	PRJNA622642	China (FJ)	Tomato	Bacterial wilt
*R. solanacearum* FJAT15249F50	NZ_CP052106	PRJNA622642	China (FJ)	Tomato	Bacterial wilt
*R. solanacearum* FJAT15252F1	NZ_CP052104	PRJNA622642	China (FJ)	Tomato	Bacterial wilt
*R. solanacearum* FJAT15252F50	NZ_CP052102	PRJNA622642	China (FJ)	Tomato	Bacterial wilt
*R. solanacearum* FJAT15304F1	NZ_CP052100	PRJNA622642	China (FJ)	Tomato	Bacterial wilt
*R. solanacearum* FJAT15304F50	NZ_CP052098	PRJNA622642	China (FJ)	Tomato	Bacterial wilt
*R. solanacearum* FJAT15304F6	NZ_CP052096	PRJNA622642	China (FJ)	Tomato	Bacterial wilt
*R. solanacearum* FJAT15340F1	NZ_CP052094	PRJNA622642	China (FJ)	Tomato	Bacterial wilt
*R. solanacearum* FJAT15340F50	NZ_CP052092	PRJNA622642	China (FJ)	Tomato	Bacterial wilt
*R. solanacearum* FJAT15340F6	NZ_CP052090	PRJNA622642	China (FJ)	Tomato	Bacterial wilt
*R. solanacearum* FJAT15353F1	NZ_CP052088	PRJNA622642	China (FJ)	Tomato	Bacterial wilt
*R. solanacearum* FJAT15353F50	NZ_CP052086	PRJNA622642	China (FJ)	Tomato	Bacterial wilt
*R. solanacearum* FJAT15353F8	NZ_CP052084	PRJNA622642	China (FJ)	Tomato	Bacterial wilt
*R. solanacearum* FJAT442F1	NZ_CP052082	PRJNA622642	China (FJ)	Tomato	Bacterial wilt
*R. solanacearum* FJAT442F50	NZ_CP052080	PRJNA622642	China (FJ)	Tomato	Bacterial wilt
*R. solanacearum* FJAT445F1	NZ_CP052078	PRJNA622642	China (FJ)	Tomato	Bacterial wilt
*R. solanacearum* FJAT445F50	NZ_CP052076	PRJNA622642	China (FJ)	Tomato	Bacterial wilt
*R. solanacearum* FJAT448F1	NZ_CP052074	PRJNA622642	China (FJ)	Tomato	Bacterial wilt
*R. solanacearum* FJAT448F50	NZ_CP052072	PRJNA622642	China (FJ)	Tomato	Bacterial wilt
*R. solanacearum* FJAT454F1	NZ_CP052070	PRJNA622642	China (FJ)	Tomato	Bacterial wilt
*R. solanacearum* FJAT454F501	NZ_CP060701	PRJNA622642	China (FJ)	Tomato	Bacterial wilt
*R. solanacearum* FJAT91	NZ_CP016612	PRJNA329188	China (FJ)	Tomato (healthy)	Bacterial wilt
*R. solanacearum* FJAT91F1	NZ_CP056083	PRJNA640736	China (FJ)	-	-
*R. solanacearum* FJAT91F50	NZ_CP052068	PRJNA622642	China (FJ)	Tomato	Bacterial wilt
*R. solanacearum* FJAT91F8	NZ_CP056085	PRJNA622642	China (FJ)	Tomato	Bacterial wilt
*R. solanacearum* GM1000	NC_003295 AL646057-AL646075	PRJNA13	-	*Arabdopsis thaliana*	Bacterial wilt
*R. solanacearum* HA41	NZ_CP022481	PRJNA392775	China (HB)	Peanut	Bacterial wilt
*R. solanacearum* IBSBF1503	NZ_CP012943	PRJNA297402	Brazil	Pepino	NPB (non-pathogenic to banana)
*R. solanacearum* IBSBF2571	NZ_CP026307	PRJNA431203	Brazil (SE)	Plaintain	Moko disease
*R. solanacearum* KACC10709	NZ_CP016904	PRJNA314721	South Korea (GY)	Potato	Bacterial wilt
*R. solanacearum* KACC10722	NZ_CP014702	PRJNA314571	South Korea (JE)	Potato	Bacterial wilt
*R. solanacearum* MAFF211471	NZ_AP024097	PRJDB10588	Japan (Kochi)	Ginger	Bacterial wilt
*R. solanacearum* MAFF211472	NZ_AP024157	PRJDB9507	Japan (Kyushu)	Ginger	Bacterial wilt
*R. solanacearum* MAFF211479	NZ_AP024099	PRJDB10588	Japan (Kochi)	Ginger	Bacterial wilt
*R. solanacearum* MAFF211491	NZ_AP024101	PRJDB10588	Japan (Kochi)	Ginger	Bacterial wilt
*R. solanacearum* MAFF241647	NZ_AP024105	PRJDB10588	Japan (Kochi)	Ginger	Bacterial wilt
*R. solanacearum* MAFF241648	NZ_AP024107	PRJDB10588	Japan (Kochi)	Ginger	Bacterial wilt
*R. solanacearum* MAFF301560	NZ_AP024103	PRJDB10588	Japan (Kochi)	Ginger	Bacterial wilt
*R. solanacearum* MAFF311693	NZ_AP024161	PRJDB9507	Japan (Kyushu)	Wild turmeric	Bacterial wilt
*R. solanacearum* OE11	NZ_CP009763	PRJDB4012	Japan (Kochi)	Eggplant	Bacterial wilt
*R. solanacearum* Po82	NC_017574	PRJNA66837	Mexico	Potato	Bacterial wilt/Moko disease
*R. solanacearum* PSI07	NC_014310	PRJEA50683	-	Tomato	Bacterial wilt
*R. solanacearum* RS488	NZ_CP021652	PRJNA388430	Brazil (PR)	Tomato	Bacterial wilt
*R. solanacearum* RS489	NZ_CP021766	PRJNA388980	Brazil (PR)	Tomato	Bacterial wilt
*R. solanacearum* RSCM	NZ_CP025985	PRJNA422474	China (GD)	Pumpkin	Bacterial wilt
*R. solanacearum* SEPPX05	NZ_CP021448	PRJNA379485	China (JX)	Sesame	Bacterial wilt
*R. solanacearum* SL2064	NZ_CP022798	PRJNA396777	South Korea (JE)	Potato	Bacterial wilt
*R. solanacearum* SL2312	NZ_CP022796	PRJNA396777	South Korea (JE)	Potato	Bacterial wilt
*R. solanacearum* SL2330	NZ_CP022794	PRJNA396777	South Korea (JE)	Potato	Bacterial wilt
*R. solanacearum* SL2729	NZ_CP022792	PRJNA396777	South Korea (JE)	Potato	Bacterial wilt
*R. solanacearum* SL3022	CP023016	PRJNA396777	South Korea (JE)	Potato	Bacterial wilt
*R. solanacearum* SL3103	NZ_CP022790	PRJNA396777	South Korea (JE)	Potato	Bacterial wilt
*R. solanacearum* SL3175	NZ_CP022788	PRJNA396777	South Korea (JE)	Potato	Bacterial wilt
*R. solanacearum* SL3300	NZ_CP022786	PRJNA396777	South Korea (JE)	Potato	Bacterial wilt
*R. solanacearum* SL3730	NZ_CP022784	PRJNA396777	South Korea (JE)	Potato	Bacterial wilt
*R. solanacearum* SL3755	NZ_CP022782	PRJNA396777	South Korea (JE)	Potato	Bacterial wilt
*R. solanacearum* SL3822	NZ_CP022780	PRJNA396777	South Korea (JE)	Potato	Bacterial wilt
*R. solanacearum* SL3882	NZ_CP022778	PRJNA396777	South Korea (JE)	Potato	Bacterial wilt
*R. solanacearum* T101	NZ_CP022757	PRJNA396777	South Korea (JE)	Potato	Bacterial wilt
*R. solanacearum* T11	NZ_CP022776	PRJNA396777	South Korea (JE)	Potato	Bacterial wilt
*R. solanacearum* T110	CP023012	PRJNA396777	South Korea (JE)	Potato	Bacterial wilt
*R. solanacearum* T117	NZ_CP022755	PRJNA396777	South Korea (JE)	Potato	Bacterial wilt
*R. solanacearum* T12	NZ_CP022774	PRJNA396777	South Korea (JE)	Potato	Bacterial wilt
*R. solanacearum* T25	CP023014	PRJNA396777	South Korea (JE)	Potato	Bacterial wilt
*R. solanacearum* T42	NZ_CP022772	PRJNA396777	South Korea (JE)	Potato	Bacterial wilt
*R. solanacearum* T51	NZ_CP022770	PRJNA396777	South Korea (JE)	Potato	Bacterial wilt
*R. solanacearum* T60	NZ_CP022768	PRJNA396777	South Korea (JE)	Potato	Bacterial wilt
*R. solanacearum* T78	NZ_CP022765	PRJNA396777	South Korea (JE)	Potato	Bacterial wilt
*R. solanacearum* T82	NZ_CP022763	PRJNA396777	South Korea (JE)	Potato	Bacterial wilt
*R. solanacearum* T95	NZ_CP022761	PRJNA396777	South Korea (JE)	Potato	Bacterial wilt
*R. solanacearum* T98	NZ_CP022759	PRJNA396777	South Korea (JE)	Potato	Bacterial wilt
*R. solanacearum* UW163	NZ_CP012939	PRJNA297400	Peru (NA)	Plaintain	Moko disease
*R. solanacearum* UW386	NZ_CP039339	PRJNA531204	Nigeria	Soil	-
*R. solanacearum* UW576	NZ_CP051175	PRJNA591018	Senegal	Tomato	Bacterial wilt
*R. solanacearum* UW763	NZ_CP051173	PRJNA591018	Senegal	Tomato	Bacterial wilt
*R. solanacearum* UW773	NZ_CP051171	PRJNA591018	Senegal	Tomato	Bacterial wilt
*R. solanacearum* UW774	NZ_CP051169	PRJNA591018	Senegal	Tomato	Bacterial wilt
*R. solanacearum* UY031	NZ_CP012687	PRJNA278086	Uruguay	Wild potato	Brown rot
*R. solanacearum* YC40M	NZ_CP015850	PRJNA314427	China (GD)	Galangal	Bacterial wilt
*R. solanacearum* YC45	CP011997	PRJNA286156	China (GD)	Ginger	Bacterial wilt
*R. solanacearum* YQ	NZ_CP059489	PRJNA648113	China (ZJ)	Casuarina pine	Bacterial wilt
